# Supplementary material for: The Birth-and-Death Evolution of Cytochrome P450 Genes in Bees
Source: Genome Biol Evol. 2021 Nov 30;13(12):evab261. doi: 10.1093/gbe/evab261 (PMC8670302; doi:10.1093/gbe/evab261)
Supplement: evab261_Supplementary_Data [file evab261_supplementary_data.zip › Supplementary_Information_minorrevisions.docx]

# Supplementary Information

# The birth-and-death evolution of cytochrome P450 genes in bees

Kathy Darragh^1^, David R. Nelson^2^ & Santiago R. Ramírez^1^

^1^Department of Evolution and Ecology, University of California, Davis, CA, 95616

^2^Department of Molecular Sciences, University of Tennessee, Memphis, TN, 38163

ORCID Kathy Darragh 0000-0002-9215-014X

ORCID David R. Nelson 0000-0003-0583-5421

ORCID Santiago R. Ramírez 0000-0003-1306-1315

**

***Supplementary Figure 1:***  *Full phylogeny of cytochrome P450s in bees. The phylogeny was constructed in IQ -TREE (model JTT+F+R10) using 481 amino acid sequences across ten species of bee. Bootstrap values (n=1000) are illustrated.*

***Supplementary Figure 2:*** *Relationship between sociality and P450 repertoire. We did not find a relationship between sociality and A) the number of P450s (Spearman’s rank correlation, S=93.891, p=0.5739, rho=0.218), B) the number of CYP3s (Spearman’s rank correlation, S=93.891, p=0.5739, rho=0.218), or C) the number of CYP6AS genes (Spearman’s rank correlation, S=52.719, p=0.1163, rho=0.5607). PIC, phylogenetically independent contrast.*

**

***Supplementary Figure 3:*** *Boxplots showing relationship between resin collection and P450 repertoire. We did not find a relationship between resin collection and A) the number of P450s (phylogenetic ANOVA, F=0.01008, df=1, p=0.94), B) the number of CYP3s (phylogenetic ANOVA, F=0.01006, df=1, p=0.94), or C) the number of CYP6AS genes (phylogenetic ANOVA, F=1.3433, df=1, p=0.366). PIC, phylogenetically independent contrast.*

**

***Supplementary Figure 4:*** *Distribution of instability scores across P450 clades. Instability scores from MiPhy output plotted for each clade. Clades to the left of the dotted line are considered stable, and those to the right unstable.*

**

***Supplementary Figure 5:*** *Boxplot showing MiPhy instability scores across the four CYP groups. Instability differs between the four groups (Welch one-way ANOVA test, F_3,13.6_=6.8, p=0.005). A Games-Howell post-hoc analysis revealed statistically significant increases in instability in the CYP3 group when compared with the other three groups CYP2 (7.07, 95% CI 2.54-11.6, p=*0.001*), CYP4 (5.98, 95% CI 1.26-10.7, p=0.009), and mitochondrial (7.28, 95% CI 2.71-11.9, 0.001). Mito; mitochondrial.*

**

***Supplementary Figure 6:*** *Relationship between cumulative patristic distance and the instability of a clade. When cumulative patristic distance is normalized for gene number in a clade, there is a significant correlation between clade cumulative patristic distance and clade instability (Spearman’s Correlation, rho=0.475, p=* *0.00191). CBL, cumulative branch length.*

**

***Supplementary Figure 7:*** *Boxplot showing distribution of all branches with dN/dS significantly different from background dN/dS of the clade (adjusted p value<0.05). Branches with a dN/dS>10 were removed. The dN/dS of branches found in unstable clades is significantly higher than that of stable clades (*t*-test, df=238.2, t=-4.837, p=0.000002364).*

*
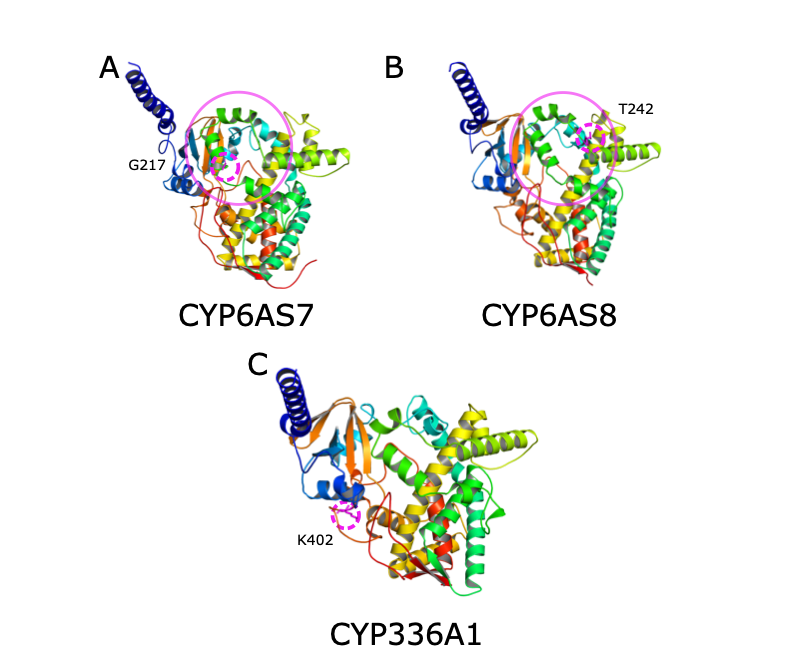
*

***Supplementary Figure 8:*** *Protein models for representative P450s for the clades where positive selection was detected in PAML site models. Structures shown for A) CYP6AS7 (GB49894), B) CYP6AS8 (GB49878) and C) CYP336A1 (GB55669). Positively selected residues are highlighted by the pink dashed line. The surface area in which residues from CYP6AS7 and CYP6AS8 are found is highlighted with the solid line circle.*

***Supplementary Table 1*:** *Results from CAFE analysis. Only clades with significant posterior probabilities are shown, full results available from OSF (*[*https://osf.io/9tdqu/?view_only=f7514c174fdb4574b77ed37ac1983f24*](https://osf.io/9tdqu/?view_only=f7514c174fdb4574b77ed37ac1983f24)*).*

| #FamilyID | Gamma Cat Median | Likelihood of Category | Likelihood of Family | Posterior Probability | Significant |
| --- | --- | --- | --- | --- | --- |
| CYP336A | 0.0170496 | 3.98E-24 | 5.92E-11 | 6.72E-14 | N/S |
| CYP336A | 0.311338 | 3.07E-14 | 5.92E-11 | 0.00051822 | N/S |
| CYP336A | 2.67161 | 5.92E-11 | 5.92E-11 | 0.999482 | * |
| CYP9DN1 | 0.0170496 | 5.24E-28 | 4.81E-10 | 1.09E-18 | N/S |
| CYP9DN1 | 0.311338 | 6.71E-16 | 4.81E-10 | 1.40E-06 | N/S |
| CYP9DN1 | 2.67161 | 4.81E-10 | 4.81E-10 | 0.999999 | * |
| CYP9R | 0.0170496 | 5.59E-21 | 7.46E-10 | 7.50E-12 | N/S |
| CYP9R | 0.311338 | 6.91E-12 | 7.46E-10 | 0.00926094 | N/S |
| CYP9R | 2.67161 | 7.39E-10 | 7.46E-10 | 0.990739 | * |
| CYP9Q | 0.0170496 | 1.15E-21 | 1.55E-09 | 7.45E-13 | N/S |
| CYP9Q | 0.311338 | 2.46E-12 | 1.55E-09 | 0.0015898 | N/S |
| CYP9Q | 2.67161 | 1.54E-09 | 1.55E-09 | 0.99841 | * |
| CYP6AQ | 0.0170496 | 3.91E-29 | 8.73E-11 | 4.47E-19 | N/S |
| CYP6AQ | 0.311338 | 5.27E-17 | 8.73E-11 | 6.03E-07 | N/S |
| CYP6AQ | 2.67161 | 8.73E-11 | 8.73E-11 | 0.999999 | * |
| CYP6AS1 | 0.0170496 | 2.79E-42 | 3.85E-13 | 7.25E-30 | N/S |
| CYP6AS1 | 0.311338 | 4.68E-22 | 3.85E-13 | 1.21E-09 | N/S |
| CYP6AS1 | 2.67161 | 3.85E-13 | 3.85E-13 | 1 | * |
| CYP6AS8 | 0.0170496 | 1.18E-21 | 5.51E-10 | 2.14E-12 | N/S |
| CYP6AS8 | 0.311338 | 2.63E-13 | 5.51E-10 | 0.00047735 | N/S |
| CYP6AS8 | 2.67161 | 5.51E-10 | 5.51E-10 | 0.999523 | * |
| CYP6AS4 | 0.0170496 | 1.92E-21 | 1.44E-09 | 1.33E-12 | N/S |
| CYP6AS4 | 0.311338 | 4.53E-13 | 1.44E-09 | 0.000315 | N/S |
| CYP6AS4 | 2.67161 | 1.44E-09 | 1.44E-09 | 0.999685 | * |

***Supplementary Table 2*:** *Results from codeml analysis of all clades comparing “one-ratio” and “free-ratio” models. Log likelihood (lnL1 for “one-ratio”, lnL2 for “free-ratio”), as well as number of parameters (np1 for “one-ratio”, np2 for “free-ratio”) are shown for both models. Twice the difference in log likelihood score (2*∆lnL) is used along with the difference in number of parameters (∆np) as the degrees of freedom to carry out a chi-squared test to compare the models. The adjusted p-value (adj. p-val) is corrected using false-detection rate correction.*

| Clade | Type | lnL1 | np1 | lnL2 | np2 | 2*∆Lnl | ∆np | adj. p-val |
| --- | --- | --- | --- | --- | --- | --- | --- | --- |
| 1 | Stable | -8885.5211 | 19 | -8877.0357 | 35 | 16.970904 | 16 | NS |
| 2 | Stable | -7595.8343 | 19 | -7576.6176 | 35 | 38.43349 | 16 | 0.00194 |
| 3 | Stable | -4276.0555 | 19 | -4255.8985 | 35 | 40.31409 | 16 | 0.00117 |
| 4 | Stable | -7277.4506 | 19 | -7256.9549 | 35 | 40.991366 | 16 | 0.000969 |
| 5 | Stable | -5443.2808 | 19 | -5433.1258 | 35 | 20.310038 | 16 | NS |
| 6 | Stable | -8222.5491 | 19 | -8212.1133 | 35 | 20.871602 | 16 | NS |
| 7 | Stable | -6158.7492 | 19 | -6146.4211 | 35 | 24.656106 | 16 | NS |
| 8 | Stable | -5894.6557 | 19 | -5882.1418 | 35 | 25.027812 | 16 | NS |
| 9 | Stable | -8893.0525 | 19 | -8875.5863 | 35 | 34.932424 | 16 | 0.00524 |
| 10 | Stable | -9143.5099 | 17 | -9118.719 | 31 | 49.581888 | 14 | 1.43E-05 |
| 11 | Stable | -9995.6908 | 19 | -9976.1735 | 35 | 39.034538 | 16 | 0.00165 |
| 12 | Stable | -5551.5747 | 17 | -5530.6696 | 31 | 41.810198 | 14 | 0.000241 |
| 13 | Stable | -6872.4131 | 19 | -6824.2831 | 35 | 96.260048 | 16 | 5.33E-13 |
| 14 | Stable | -7565.7418 | 19 | -7548.038 | 35 | 35.407436 | 16 | 0.00466 |
| 15 | Stable | -9248.6049 | 17 | -9200.8067 | 31 | 95.59643 | 14 | 1.20E-13 |
| 16 | Stable | -7084.2574 | 17 | -7066.1734 | 31 | 36.16804 | 14 | 0.00158 |
| 17 | Stable | -8112.4327 | 19 | -8066.5746 | 35 | 91.71616 | 16 | 3.22E-12 |
| 18 | Stable | -7385.8277 | 15 | -7357.9912 | 27 | 55.672872 | 12 | 3.22E-07 |
| 19 | Stable | -8588.996 | 19 | -8539.4733 | 35 | 99.045384 | 16 | 1.74E-13 |
| 20 | Unstable | -13023.38 | 59 | -12939.732 | 115 | 167.2961 | 56 | 1.46E-12 |
| 21 | Stable | -4553.6841 | 5 | -4553.233 | 7 | 0.902394 | 2 | NS |
| 22 | Stable | -6548.3633 | 9 | -6541.8329 | 15 | 13.060756 | 6 | NS |
| 23 | Stable | -8640.8138 | 15 | -8599.5453 | 27 | 82.537118 | 12 | 3.38E-12 |
| 24 | Stable | -6546.0139 | 15 | -6531.0135 | 27 | 30.000666 | 12 | 0.00385 |
| 25 | Unstable | -15955.605 | 29 | -15842.428 | 55 | 226.355292 | 26 | 7.25E-33 |
| 26 | Stable | -8377.5506 | 13 | -8368.5158 | 23 | 18.069706 | 10 | NS |
| 27 | Unstable | -21124.722 | 51 | -20935.455 | 99 | 378.53361 | 48 | 2.63E-51 |
| 28 | Unstable | -20864.274 | 39 | -20736.596 | 75 | 255.355564 | 36 | 9.76E-34 |
| 29 | Stable | -10640.314 | 19 | -10609.684 | 35 | 61.260314 | 16 | 6.75E-07 |
| 30 | Unstable | -16679.891 | 35 | -16552.376 | 67 | 255.02871 | 32 | 2.77E-35 |
| 31 | Stable | -8462.2254 | 21 | -8442.6119 | 39 | 39.226992 | 18 | 0.00379 |
| 32 | Stable | -7188.2349 | 19 | -7163.8779 | 35 | 48.714076 | 16 | 6.98E-05 |
| 33 | Unstable | -14376.647 | 33 | -14303.319 | 63 | 146.657174 | 30 | 1.34E-16 |
| 34 | Unstable | -17683.192 | 69 | -17560.453 | 135 | 245.476706 | 66 | 1.19E-21 |
| 35 | Stable | -8131.8418 | 15 | -8125.2231 | 27 | 13.237244 | 12 | NS |
| 36 | Unstable | -15279.119 | 29 | -15214.61 | 55 | 129.01923 | 26 | 5.11E-15 |
| 37 | Unstable | -11734.964 | 27 | -11697.211 | 51 | 75.505834 | 24 | 6.75E-07 |
| 38 | Stable | -9591.5457 | 23 | -9512.0793 | 43 | 158.932784 | 20 | 9.65E-23 |
| 39 | Unstable | -10903.749 | 25 | -10840.505 | 47 | 126.48877 | 22 | 5.07E-16 |
| 40 | Unstable | -9711.623 | 21 | -9651.1106 | 39 | 121.024766 | 18 | 1.34E-16 |

***Supplementary Table 3*:** *Results from codeml analysis of all clades comparing M7 and M8 models to test for positively selected sites. Log likelihood (lnL1 for “one-ratio”, lnL2 for “free-ratio”), as well as number of parameters (np1 for M7, np2 for M8 are shown for both models. Twice the difference in log likelihood score (2*∆lnL) is used along with the difference in number of parameters (∆np) as the degrees of freedom to carry out a chi-squared test to compare the models. The adjusted p-value (adj. p-val) is corrected using false-detection rate correction. Results shown are those from the PRANK alignments as these were more stringent, resulting in fewer clades under positive selection, results for results with MAFFT alignments are in Supplementary data 3.*

| Clade | Type | lnL1 | np1 | lnL2 | np2 | 2*∆Lnl | ∆np | adj. p-val |
| --- | --- | --- | --- | --- | --- | --- | --- | --- |
| 1 | Stable | -8729.1682 | 20 | -8729.1697 | 22 | -0.0029 | 2 | NS |
| 2 | Stable | -7447.1223 | 20 | -7445.4071 | 22 | 3.430492 | 2 | NS |
| 3 | Stable | -4057.4928 | 20 | -4056.339 | 22 | 2.307438 | 2 | NS |
| 4 | Stable | -7101.1446 | 20 | -7098.928 | 22 | 4.43323 | 2 | NS |
| 5 | Stable | -5357.0905 | 20 | -5356.5521 | 22 | 1.076818 | 2 | NS |
| 6 | Stable | -7955.1546 | 20 | -7955.1541 | 22 | 0.000986 | 2 | NS |
| 7 | Stable | -5815.7835 | 20 | -5815.786 | 22 | -0.005102 | 2 | NS |
| 8 | Stable | -5834.2268 | 20 | -5831.4562 | 22 | 5.54115 | 2 | NS |
| 9 | Stable | -8424.8757 | 20 | -8422.9156 | 22 | 3.920212 | 2 | NS |
| 10 | Stable | -8958.4551 | 18 | -8956.6764 | 20 | 3.557374 | 2 | NS |
| 11 | Stable | -9800.6502 | 20 | -9796.7645 | 22 | 7.77153 | 2 | NS |
| 12 | Stable | -5343.4895 | 18 | -5339.6426 | 20 | 7.693912 | 2 | NS |
| 13 | Stable | -6568.5908 | 20 | -6566.5626 | 22 | 4.056502 | 2 | NS |
| 14 | Stable | -7506.351 | 20 | -7506.3516 | 22 | -0.001262 | 2 | NS |
| 15 | Stable | -8896.2614 | 18 | -8894.432 | 20 | 3.658748 | 2 | NS |
| 16 | Stable | -7006.6508 | 18 | -7006.544 | 20 | 0.213514 | 2 | NS |
| 17 | Stable | -7948.314 | 20 | -7942.1171 | 22 | 12.39376 | 2 | 0.0156 |
| 18 | Stable | -6915.1618 | 16 | -6912.3721 | 18 | 5.57947 | 2 | NS |
| 19 | Stable | -8490.8154 | 20 | -8488.0952 | 22 | 5.440374 | 2 | NS |
| 20 | Unstable | -12605.842 | 60 | -12596.921 | 62 | 17.841794 | 2 | 0.00267 |
| 21 | Stable | -4509.4506 | 6 | -4508.5706 | 8 | 1.760018 | 2 | NS |
| 22 | Stable | -6430.1841 | 10 | -6429.1908 | 12 | 1.986708 | 2 | NS |
| 23 | Stable | -8492.2938 | 16 | -8490.4168 | 18 | 3.754064 | 2 | NS |
| 24 | Stable | -6371.2304 | 16 | -6371.2308 | 18 | -0.000928 | 2 | NS |
| 25 | Unstable | -15532.387 | 30 | -15532.293 | 32 | 0.18685 | 2 | NS |
| 26 | Stable | -8293.0265 | 14 | -8292.8529 | 16 | 0.34709 | 2 | NS |
| 27 | Unstable | -20321.076 | 52 | -20316.172 | 54 | 9.808274 | 2 | 0.0371 |
| 28 | Unstable | -20211.944 | 40 | -20211.838 | 42 | 0.211592 | 2 | NS |
| 29 | Stable | -10337.078 | 20 | -10330.211 | 22 | 13.73349 | 2 | 0.0104 |
| 30 | Unstable | -16308.102 | 36 | -16304.977 | 38 | 6.25095 | 2 | NS |
| 31 | Stable | -8278.2587 | 22 | -8277.4946 | 24 | 1.528132 | 2 | NS |
| 32 | Stable | -7171.6122 | 20 | -7171.615 | 22 | -0.005636 | 2 | NS |
| 33 | Unstable | -14099.503 | 34 | -14098.449 | 36 | 2.108006 | 2 | NS |
| 34 | Unstable | -17119.494 | 70 | -17108.921 | 72 | 21.146588 | 2 | 0.00102 |
| 35 | Stable | -7982.0468 | 16 | -7982.0482 | 18 | -0.00277 | 2 | NS |
| 36 | Unstable | -14941.539 | 30 | -14935.481 | 32 | 12.115786 | 2 | 0.0156 |
| 37 | Unstable | -11314.303 | 28 | -11312.278 | 30 | 4.050414 | 2 | NS |
| 38 | Stable | -9429.9085 | 24 | -9422.8252 | 26 | 14.166712 | 2 | 0.0104 |
| 39 | Unstable | -10613.879 | 26 | -10612.795 | 28 | 2.16674 | 2 | NS |
| 40 | Unstable | -9527.1926 | 22 | -9521.5805 | 24 | 11.224162 | 2 | 0.0209* |

*This clade was not identified as positively selected in the MAFFT alignment results.
